# CRISPLD2 (LGL1) inhibits proinflammatory mediators in human fetal, adult, and COPD lung fibroblasts and epithelial cells

**DOI:** 10.14814/phy2.12942

**Published:** 2016-09-05

**Authors:** Hui Zhang, Alvin T. Kho, Qing Wu, Andrew J. Halayko, Karen Limbert Rempel, Robert P. Chase, Neil B. Sweezey, Scott T. Weiss, Feige Kaplan

**Affiliations:** ^1^ Research Institute of the McGill University Health Centre Montreal Quebec Canada; ^2^ Children's Hospital Informatics Program Boston Children's Hospital Boston Massachusetts; ^3^ Department of Physiology and Pathophysiology University of Manitoba Winnipeg Manitoba Canada; ^4^ Department of Internal Medicine University of Manitoba Winnipeg Manitoba Canada; ^5^ Biology of Breathing Group Manitoba Institute of Child Health Winnipeg Manitoba Canada; ^6^ Channing Division of Network Medicine Department of Medicine Brigham and Women's Hospital Harvard Medical School Boston Massachusetts; ^7^ The Hospital for Sick Children Research Institute Toronto Ontario Canada; ^8^ Departments of Paediatrics and Physiology University of Toronto Toronto Ontario Canada; ^9^ Departments of Human Genetics and Pediatrics McGill University Montreal Quebec Canada

**Keywords:** Bronchopulmonary dysplasia (BPD), CRISPLD2 (LGL1), human fetal/adult lung fibroblast, mesenchymal–epithelial interaction, pro/antiinflammation

## Abstract

Chronic lung disease of prematurity/bronchopulmonary dysplasia (BPD) is the leading cause of perinatal morbidity in developed countries. Inflammation is a prominent finding. Currently available interventions have associated toxicities and limited efficacy. While BPD often resolves in childhood, survivors of preterm birth are at risk for acquired respiratory disease in early life and are more likely to develop chronic obstructive pulmonary disease (COPD) in adulthood. We previously cloned Crispld2 (Lgl1), a glucocorticoid‐regulated mesenchymal secretory protein that modulates lung branching and alveogenesis through mesenchymal–epithelial interactions. Absence of *Crispld2* is embryonic lethal. Heterozygous *Crispld2+/−* mice display features of BPD, including distal airspace enlargement, disruption of elastin, and neonatal lung inflammation. CRISPLD2 also plays a role in human fetal lung fibroblast cell expansion, migration, and mesenchymal–epithelial signaling. This study assessed the effects of endogenous and exogenous CRISPLD2 on expression of proinflammatory mediators in human fetal and adult (normal and COPD) lung fibroblasts and epithelial cells. CRISPLD2 expression was upregulated in a lipopolysaccharide (LPS)‐induced human fetal lung fibroblast line (MRC5). LPS‐induced upregulation of the proinflammatory cytokines IL‐8 and CCL2 was exacerbated in MRC5‐*CRISPLD2* ^knockdown^ cells. siRNA suppression of endogenous *CRISPLD2* in adult lung fibroblasts (HLFs) led to augmented expression of IL‐8, IL‐6, CCL2. LPS‐stimulated expression of proinflammatory mediators by human lung epithelial HAEo‐ cells was attenuated by purified secretory CRISPLD2. RNA sequencing results from HLF‐*CRISPLD2* ^knockdown^ suggest roles for CRISPLD2 in extracellular matrix and in inflammation. Our data suggest that suppression of CRISPLD2 increases the risk of lung inflammation in early life and adulthood.

## Introduction

Preterm birth affects 12.5% of pregnancies in the United States, and this rate continues to increase (Goldenberg et al. 2008). Chronic lung disease of infancy‐bronchopulmonary dysplasia (BPD) affects up to 43% of infants born at <1500 g (Stoll et al. 2010). BPD is the leading cause of perinatal morbidity in developed countries. While BPD often resolves in childhood, survivors of preterm birth require increased hospitalization for acquired respiratory disease in early life and are more likely to develop chronic obstructive pulmonary disease (COPD) in adulthood (Kramer et al. 2009). Unfortunately, for the prevention or treatment of BPD, the few interventions currently available (such as mechanical ventilation, oxygen therapy, nutritional support, and corticosteroid therapy) are of limited efficacy (Allen et al. 2003; Wright and Kirpalani 2011) and are associated with significant toxicities.

The prominent pulmonary pathological manifestations of infants dying from BPD are: arrested alveogenesis, airway inflammation, and parenchymal fibrosis (Jobe and Bancalari 2001). Lung inflammation associated with prenatal chorioamnionitis and postnatal respiratory tract infections is associated with disease progression (Allen et al. 2003). Proinflammatory cyto‐/chemokine factors, present in the air spaces of ventilated preterm infants, are increased in the air spaces of infants who subsequently develop BPD. In a prospective cohort study of 1067 preterm infants, of whom 606 developed BPD, an early increase in IL‐8 and IL‐6 levels predicted BPD (Ambalavanan et al. 2009). In transgenic mice, overexpression of the proinflammatory mediators TNF‐*α*, TNF‐*β*, IL‐6, and IL‐1*β* interferes with alveolarization, suggesting that the proinflammatory environment of the air space of the preterm infant may contribute to the arrest in BPD of the secondary septation involved in alveolar formation (Jobe and Bancalari 2001). Although inflammation is essential to the pathogenesis of BPD, to this date, this understanding has not translated into useful therapies (Watterberg 2010; Wright and Kirpalani 2011). Glucocorticoids (GCs) have serious side effects, including aggravation of pulmonary developmental arrest, gastrointestinal perforation, cardiac hypertrophy, short‐ and long‐term systemic growth failure, and serious neurodevelopmental delay (Jobe and Bancalari 2001; Bassler et al. 2015). A better understanding of the inflammatory regulators involved in the pathogenesis of the disease will be important in finding effective new therapies for BPD.

We previously identified and cloned CRISPLD2 (which we originally called late gestation lung 1, LGL1), a GC‐ and developmentally regulated gene encoding a secreted mesenchymal protein in the lung and other organs (Himes et al. 2014). CRISPLD2 modulates both airway branching morphogenesis and alveogenesis through mesenchymal–epithelial interactions (Oyewumi et al. 2003b; Nadeau et al. 2006; Lan et al. 2009). Expression of *Crispld2* can be directly upregulated by GC in primary rat fetal lung fibroblasts (Kaplan et al. 1999). Absence of *Crispld2* in null mice is lethal in early embryogenesis. Heterozygous *Crispld2*+/− mice have features that resemble human BPD, including distal airspace enlargement, disruption of elastin, and signs of neonatal lung inflammation (including goblet cell hyperplasia) and elevated expression of proinflammatory mediators (Lan et al. 2009).

Lung fibroblasts are involved in developmental airway remodeling as well as in tissue repair following inflammatory injury of small airways (Chapman 2011). Recently, we showed that CRISPLD2 regulates lung fibroblast proliferation and apoptosis, and lung epithelial cell migration through mesenchymal–epithelial interactions. These findings confirmed a functional role for CRISPLD2 in lung epithelial maturation (Zhang et al. 2015). In this study we explored the role of CRISPLD2 in inflammation in fetal and adult lung fibroblasts. CRISPLD2 is a cystine‐rich secreted protein (CRISP), characterized by a secretory signal and two LCCL domains. The LCCL domain, previously identified in horseshoe crab *Limulus* factor, binds to lipopolysaccharide (LPS, the immunostimulatory component of gram‐negative bacteria), is protective against endotoxin, and is involved in antibody‐independent host defense (Trexler et al. 2000; Gibbs et al. 2008).

This study used genetic silencing of CRISPLD2 and recombinant CRISPLD2 (rCRISPLD2) protein to investigate the effect of endogenous and exogenous CRISPLD2 on the expression and secretion of the proinflammatory cyto/chemokines IL‐8 and CCL2 by human fetal lung fibroblasts (MRC‐5), adult primary lung fibroblasts (HLF), and airway epithelial (HAEo‐) cells. Our findings suggest that CRISPLD2 serves as an endogenous antiinflammatory gene in lung fibroblasts that also curtails proinflammatory signaling by lung epithelial cells through mesenchymal–epithelial interactions. These data support the hypothesis that inhibition of CRISPLD2 increases the risk of lung inflammation in early life and in adulthood.

To begin to explore the potential role of CRISPLD2 in adult lung disease, we investigated CRISPLD2 expression in lung fibroblasts isolated from controls and from COPD patients. Recent advances in sequencing technologies have made possible comprehensive and in‐depth characterization of transcriptomes using RNA‐seq (Ozsolak and Milos 2011). We therefore used RNA‐seq to characterize transcriptomic changes following knockdown of CRISPLD2 in each of the cell lines. We identified differentially expressed genes in normal and in COPD lung fibroblasts representing functional categories including cellular response to hormone stimulation (steroid hormone and insulin signaling pathway, cAMP mediated pathway), chemokine signaling pathway, development, regulation of cell migration, and extracellular matrix regulation.

## Materials and Methods

### Lentiviral transduction of short hairpin RNA (shRNA) CRISPLD2 in MRC‐5 cells

Human fetal lung fibroblasts, MRC5 (ATCC, Manassas, VA), grown in minimal essential medium (MEM) (Wisent, Montreal, QC, Canada) with 10% (v/v) FBS and 1% penicillin/streptomycin, were used between passages 5–15, such that studies were completed before the MRC‐5 cells enter senescence. *CRISPLD2* shRNA constructs, and a scrambled shRNA used as a negative control, were cloned into a pTRIPZ lentiviral doxycycline (DOX)‐inducible vector (Thermo, Ottawa, ON, Canada). Lentivirus was produced by transfecting 293T cells with shRNA constructs pVSV‐G and psPAX2 using lipofectamine 2000 (Life technology, Burlington, ON, Canada). MRC‐5 cells were infected with lentivirus and puromycin (0.5 *μ*g/mL) (Wisent) was added after 48 h to select stable clones. The characterization of MRC5^CRISPLD2KD^ has been described in detail previously by Zhang et al. (2015). Suppression of CRISPLD2 protein levels in MRC5^CRISPLD2KD^ cells after 4 days DOX induction was confirmed by western blot analysis. CRISPLD2 was suppressed by DOX induction only in MRC5^CRISPLD2KD^ and not in MRC5^control^ cells. All further experiments were carried out using 2 *μ*g/mL DOX induction for 4 days in MRC5^CRISPLD2KD^ cells. The suppression of *CRISPLD2* mRNA in MRC5^CRISPLD2KD^ cultures was repeatedly confirmed prior to each set of experiments.

### Primary human lung fibroblast cell culture and siRNA transfection

Primary cultured human airway fibroblasts (HLF) were prepared from second to fourth generation peripheral lung tissue in resected specimens. All procedures were approved by the Human Research Ethics Board (University of Manitoba) and all donors gave informed consent. As described in detail (Ghavami et al. 2010; Sharma et al. 2015), after microdissection to separate the lamina reticularis and submucosal compartment from encircling airway smooth muscle bundle, HLF were isolated by enzymatic dissociation. For all experiments, passage 3–7 of HLF were used and cells were cultured in fibroblast medium (Sciencell Research Laboratories, Carlsbad, CA). The medium was changed every 48 h. Transfection of *CRISPLD2* siRNA and nontargeting siRNA (negative control) (siRNA universal non‐targeting control 1, Sigma‐Aldrich Corporation, St. Louis, MO) was performed using DharmaFECT 1 reagent according to the manufacturer's recommended protocol (Thermo Scientific, Lafayette, CO). The final concentration of siRNA was 25 nmol/L; siRNA sequences for CRISPLD2 knockdown were as follows: 5′‐GAACCAACAUCUAUGCAGA(dT)(dT)‐3′ and 5′‐UCUGCAUAGAUCUUGGUUC(dT)(dT)‐3. Primary HLF cells from five normal and five COPD patients (GOLD classification) are used for siRNA transfection. The suppression of *CRISPLD2*mRNA in HLF cultures was confirmed prior to each set of experiments.

### RNA‐seq library construction and sequencing

Total RNA was extracted from normal (Norm) and COPD HLF cells using the miRNAeasy mini kit (QIAGEN Inc.,Toronto, ON). A quantity of 1 mg of RNA from each sample was used to generate RNA‐seq cDNA libraries for sequencing using TruSeq Stranded Total RNA Library Prep Kit (Illumina Inc., San Diego, CA).

### RNA‐seq data analysis

We performed RNA‐seq transcriptome profiling of fibroblasts from four normal (age: 65–77 years, male) and four COPD human patients (age: 58–68 years, male, moderate‐severe COPD, GOLD classification), which are individually transfected with siRNA negative control or CRISPLD2 knockdown, in technical duplicates. The reads from resulting RNA‐seq output FASTQ files were trimmed of the TruSeq adapter sequences using Skewer (Jiang et al. 2014) with default parameters. FastQC (http://www.bioinformatics.babraham.ac.uk/projects/fastqc/) was used to visualize Phred quality scores for initial quality control. The trimmed reads were aligned against the primary assembly of the human genome version GRCh38 (http://www.ncbi.nlm.nih.gov/projects/genome /assembly/grc/human/) concatenated with ERCC RNA Spike‐In mix 1 (Thermo Fisher Scientific Inc, Waltham, MA) FASTA files using STAR aligner (Dobin et al. 2013) to create a BAM file (https://samtools.github.io/hts-specs/SAMv1.pdf) for each sample. Mapping rates and other quality control metrics suggested a high level of sequencing quality. The CuffNorm tool in Cufflinks (Trapnell et al. 2012) was used to synthesize the BAM files into the count data matrix D of 65,972 genes ×32 samples. We performed subsequent bioinformatics analyses on the quantile normalized data matrix log2(D + 10). Wilcoxon rank‐sum test was used to assess differential expression of a gene between negative control and CRISPLD2 knockdown samples in the healthy and COPD patient groups separately. A gene was considered significantly differentially expressed when its rank‐sum *P*‐value was <0.05 and the log2 fold change magnitude >1 (twofold in linear scale). Gene ontology analysis of sets of significantly differentially expressed genes was performed using DAVID 6.7(da Huang et al. 2009) .

### CRISPLD2‐open reading frame (ORF) protein purification

#### Cloning and recombinant expression of human *CRISPLD2* in HEK293 cells

The human *CRISPLD2* ORF cDNA was cloned into the vector pIRES2 (Clontech, Palo Alto, CA) with the green fluorescent protein (GFP) and V5 and His tags. GFP not fused to the inserted protein allows monitoring of transfection efficiency. V5 fused to the inserted protein before the stop codon allows for identification of the inserted protein. His tag is fused to the inserted protein after V5 and before the stop codon. HEK293 cells were transfected with human *CRISPLD2* ORF plasmid or empty vector using lipofectamine 2000. After 48 h, G418 600 *μ*g/mL was added for selection. After 2 weeks, stable cell clones from single cells were used. Protein expression of CRISPLD2 in HEK293^control^ (containing empty vector) and HEK293^CRISPLD2ORF^ cell lysates and media were examined by western blot using the V5 antibody as previously reported by Zhang et al. (2015). The stable cell line HEK293‐CRISPLD2‐HIS‐V5 was maintained in Dulbecco's modified eagle's medium (DMEM) with 10% (v/v) heat‐inactivated (56°C 30 min) fetal bovine serum, 1% penicillin/streptomycin, and 600 *μ*g/mL G418 (Life Technologies, Carlsbad, CA). The cells were passaged 1:3 when they reached 95% density. The cells were expanded into three 225 cm^2^ Corning Flasks (Corning Inc., Acton, MA) to get enough supernatant for purification. When they were 80% confluent, DMEM (serum‐free, antibiotic‐free) medium was added carefully after washing the flasks twice with PBS, and cultured for 48 h. Cell culture supernatant was collected for detection and purification.

#### Preparation of rCRISPLD2

Cell culture broth was centrifuged and loaded onto Ni SephoroseTM6 Fast Flow 0.5 mL (GE, Mississauga, ON, Canada) at 1.0 mL/min. After washing and elution with appropriate buffer, the eluted fractions were pooled and buffer exchanged to PBS, pH = 7.2. The purified protein was analyzed by SDS‐PAGE and western blot.

#### Purified protein activity assessment by migration assay

Transwell migration assays were performed according to the manufacturer's protocol (BD Bioscience, Mississauga, ON, Canada). The human tracheal epithelial cell line (1HAEo‐) was a kind gift from Dr. Dieter Gruenert (University of California at San Francisco) (Cozens et al. 1992). 1HAEo‐ cells were grown in MEM medium containing 2 mmol/L L‐glutamine with 10% (v/v) FBS and 1% penicillin/streptomycin. For the migration assay, serum‐free medium containing 1.25 × 10^4^ 1HAEo‐ cells were placed on transwell membranes (8.0 *μ*m). The transwell inserts were placed in a 24‐well plate filled with medium containing various amounts of rCRISPLD2 for 18 h. Conditioned medium collected from HEK293^CRISPLD2ORF^ were used as positive control (PC). Medium without rCRISPLD2 was used as negative control. Cells that passed through the membranes were fixed, stained and then assessed and counted under light microscopy. Each condition was tested in three separate wells of three individual experiments. For each experiment, cell viability was assessed by trypan blue exclusion before use in assays.

### Real‐time quantitative polymerase chain reaction (qRT‐PCR)

mRNA was assessed by qRT‐PCR as described (Zhang et al. 2015). Briefly, RNA was isolated from cells using Trizol (Life Technology) according to the manufacturer's protocol. Quantitative real‐time PCR was performed on the LightCycler 480 (Roche, Indianapolis, IN) with SYBR green master mix (Roche). Gene‐specific primers used for SYBR green detection of genes were as follows: Human CRISPLD2‐F, CCATGTTTGGCTCCAACCGA; Human CRISPLD2‐R, TGGGCAC TGGTACCTGTTACA; Human IL‐8‐F, GAGTGGACCACACTGCGCCAA; Human IL‐8‐R, TCCACAACCCTCTGCACCCAGTT; Human IL‐6‐F, ATTCGGTACATCCT CGAC; Human Human IL‐6‐R, TGATGATTTTCACCAGGC; Human CCL‐2‐F, CTCATAGCA GCCACCTTCAT; Human CCL‐2‐R, TGCTGCTGGTGATTCTTCTA, Human *GAPDH*‐F, GAGTCCACTGGCGTCTTCA; Human *GAPD*H‐R, GGGGTGCTAAGCAGTTGGT. *GAPDH* was used as a housekeeping gene. Relative mRNA expression levels were analyzed using the ΔΔ cycle threshold method (*n *≥* *4 for all assays).

### Immunoblotting

Proteins in cell lysate and supernatants (50 *μ*g) were separated by SDS‐PAGE and transferred to PVDF membranes. Primary antibodies rabbit anti‐CRISPLD2 (1:1000, Thermo Fisher Scientific) and rabbit‐anti‐actin (1:6000, Santa Cruz Biotechnology, Dallas, TX) were used as previously described by Zhang et al. (2015).

### ELISA

IL‐8 secretion in cell‐free supernatants was assessed with a commercial human IL8 ELISA kit (R&Dsystems, Minneapolis, MN) according to the manufacturer's instructions.

### Statistical analysis

All results are presented as mean ± SEM. One‐way analysis of variance (ANOVA), followed by post hoc analysis with Tukey's test was used for determining the significance of the differences in mean values among more than two groups. Two‐way ANOVA was used, as stated in the Figure Legends, for two factors comparison. Student's *t* test was used to compare only one factor between two groups (Prism, GraphPad, La Jolla, CA). Statistical differences were considered significant at *P *<* *0.05.

## Results

### CRISPLD2 expression in MRC5 cells is increased after LPS stimulation

**Figure 1:**
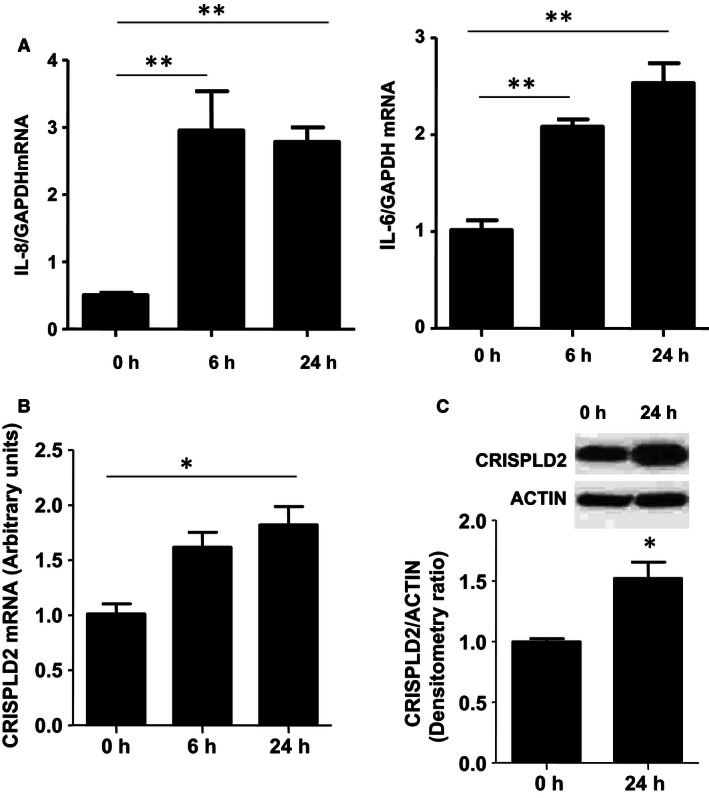
Lipopolysaccharide (LPS) stimulation increased the expression of CRISPLD2 in MRC‐5 cells. MRC‐5 cells were treated by LPS (1 *μ*g/mL) for 6 h and 24 h. Cells were harvested at indicated time points and mRNA expression of *IL‐8* and *IL‐6* (A), and *CRISPLD2* (B), were determined by q‐PCR and normalized to *GAPDH* (*n* = 4,*P < 0.05, **P <* *0.01 vs. 0 h). C: CRISPLD2 protein levels at 0 h and 24 h after LPS treatment were determined by western blot with anti‐CRISPLD2 antibody, and normalized to ACTIN (*n *= 3,* *P *<* *0.05).

LPS induces lung inflammation (Eutamene et al. 2005). To determine if LPS induces inflammation in fetal lung fibroblasts, MRC‐5 cells were treated with LPS at 1 *μ*g/mL for 6 h and 24 h. Levels of IL‐6 and IL‐8, examined by q‐PCR, were significantly upregulated after LPS treatment, suggesting that LPS can induce inflammation in MRC‐5 cells (Fig. 1A). Under the same conditions, significant upregulation of CRISPLD2 at both transcriptional and protein levels by LPS was shown by q‐PCR (Fig. 1B) and western blot (Fig. 1C), suggesting a role for CRISPLD2 in fetal lung fibroblast inflammation.

### Suppression of CRISPLD2 augments LPS‐induced inflammation in MRC5 cells

**Figure 2:**
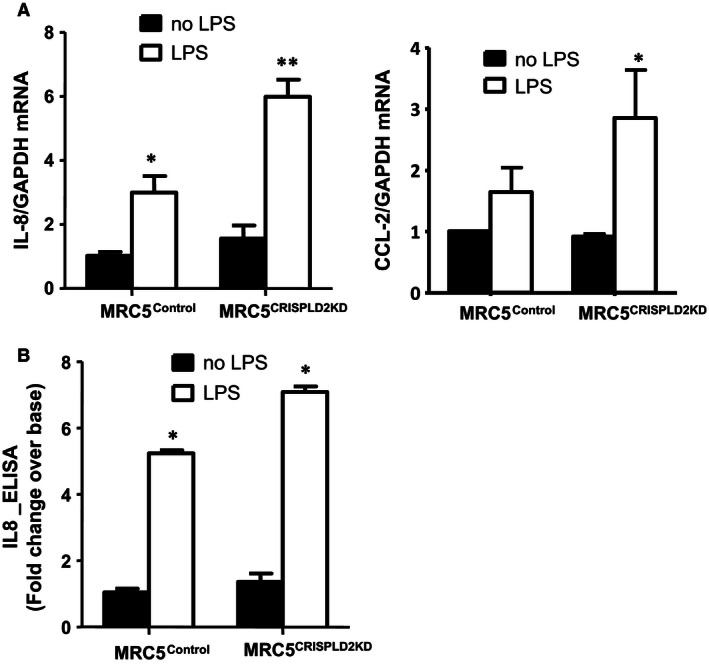
Suppression of CRISPLD2 in MRC5^CRISPLD2KD^ exacerbated lipopolysaccharide (LPS)‐induced inflammation. MRC5^CRISPLD2KD^ and MRC5^control^ were grown to 80% confluence in 6‐well plates, and treated with LPS 1 *μ*g/mL in serum‐contained medium EMEM for 24 h. Cells were collected for RNA isolation and RT‐PCR. Expression of *IL‐8* and *CCL‐2* were assessed by q‐PCR (*n* = 4, *P <* *0.05, **P <* *0.01 in treatment with LPS vs. no LPS, #*P *<* *0.05 in MRC5^CRISPLD2KD^ vs. MRC5^control^ by two‐way analysis of variance [ANOVA]). Secretion of IL‐8 into supernatant was assessed by ELISA. (*n *= 3, *P <* *0.05, fold change over the base: MRC5 ^control^ without LPS; #*P *<* *0.05 in MRC5^CRISPLD2KD^ vs. MRC5^control^ by two‐way ANOVA).

To determine how CRISPLD2 modulates LPS‐induced inflammation in human fetal lung fibroblasts, DOX‐inducible *CRISPLD2* shRNA was used to knockdown the expression of endogenous CRISPLD2 in MRC‐5. CRISPLD2 expression was reduced by DOX induction for 4 days to 30% in MRC5 ^CRISPLD2KD^ as compared with MRC5 ^control^. Proinflammatory factors *IL‐8* and *CCL‐2*, were examined in both MRC5 ^control^ and MRC5 ^CRISPLD2KD^ following LPS treatment at 1 *μ*g/mL for 24 h. As shown in Figure 2A, the increased levels of proinflammatory factors *IL‐8* and *CCL‐2* mRNA expression in MRC5^control^ cells upon LPS induction is augmented in MRC‐5^CRISPLD2KD^. By ELISA, secreted IL‐8 protein from cells was also increased in MRC5 ^control^ supernatant upon LPS induction, and this effect was augmented in MRC5 ^CRISPLD2KD^ supernatant (Fig. 2B). This result suggests that endogenous CRISPLD2 in fetal lung fibroblasts exerts an antiinflammatory effect by controlling the levels of proinflammatory cytokines.

### Recombinant CRISPLD2 inhibited LPS‐induced inflammation in human airway epithelial 1HAEo‐ cells

**Figure 3:**
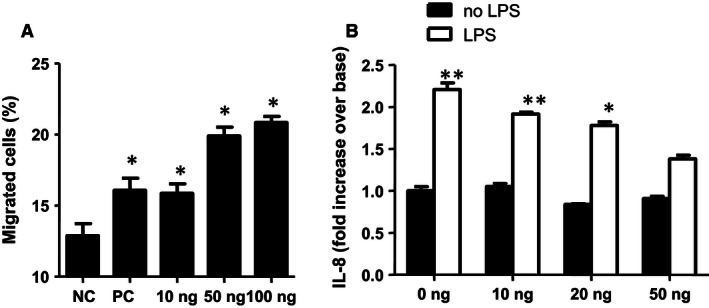
rCRISPLD2 suppressed lipopolysaccharide (LPS)‐induced inflammation in lung airway epithelial 1HAEo‐cells. (A) Activity of rCRISPLD2 after purification was evaluated by transwell migration assay. 1HAEo‐ cell migration (%) was significantly increased in the presence of rCRISPLD2 at indicated amounts in a dose‐dependent manner. CRISPLD2‐conditioned medium was used as positive control (PC), nontreated cells were used as a negative control (NC) (*n *= 3, *P <* *0.05 vs. NC). (B) 1HAEo‐ cells were treated by LPS (2.5 *μ*g/mL) for 24 h with added rCRISPLD2 at various doses. (C) By ELISA, rCRISPLD2 also inhibited LPS‐induced IL‐8 protein secretion from 1HAEo‐ in a dose‐dependent manner (*N* = 3,*P <* *0.05, **P <* *0.01 no LPS vs. LPS, two‐way analysis of variance).

Longitudinally, mesenchymal–epithelial interactions not only modulate fetal airway branching and postnatal alveogenesis in lung development but also contribute to the pathogenesis of pulmonary fibrosis and COPD in adulthood due to epithelial–mesenchymal transition (Chapman 2011). Among the mediators involved in mesenchymal–epithelial interactions in the lung, the spatial localization of CRISPLD2‐secreting myofibroblasts (Nadeau et al. 2006) points to a role for Crispld2 in fibroblast‐epithelial communication. To test whether secreted mesenchymal CRISPLD2 regulates inflammation in lung airway epithelial (1HAEo‐) cells, recombinant CRISPLD2_ORF (rCRISPLD2) protein was purified from HEK293 CRISPLD2‐conditioned medium and the activity of rCRISPLD2 after purification was validated. Assessing the dosage and toxicity of rCRISPLD2, 1HAEo‐ cells were initially incubated with rCRISPLD2 at 10, 50, 100, 125 ng, or 250 ng/mL for either 24 h or 48 h. Cell morphology and cell number were compared with untreated cells. No effect of rCRISPLD2 on cell morphology and cell death was observed in rCRISPLD2 concentrations ranging from 10 to 100 ng/mL. This dosage range was selected for further validation of protein activity, avoiding the potential toxic effects associated with higher protein dosage. We validated rCRISPLD2 protein activity by transwell migration assay. CRISPLD2‐conditioned medium promoted 1HAEo‐ cell migration through transwell membranes, consistent with our previous findings (Zhang et al. 2015). As shown in Figure 3A, 10, 50 ng, 100 ng/mL of rCRISPLD2 promotes migrated cells through the transwell membrane as compared to untreated cells in a dose‐dependent manner, supporting the preservation of protein function or activity in purified rCRISPLD2 protein. We used previously validated CRISPLD2‐conditioned medium as a positive control.

In order to evaluate the effect of rCRISPLD2 on LPS‐induced inflammation in lung airway epithelial cells, 1HAEo‐ cells were treated for 24 h with or without LPS 2.5 *μ*g/mL, plus 0, 10, 20 ng, or 50 ng/mL of rCRISPLD2. By ELISA, rCRISPLD2 inhibited LPS‐induced IL‐8 protein secretion from lung epithelial cells in a dose‐dependent manner (Fig. 3B). These results support an antiinflammatory effect of exogenous CRISPLD2 on lung epithelial cells and suggest that CRISPLD2 can reduce proinflammatory signaling by lung epithelial cells through mesenchymal–epithelial interactions.

### Knockdown of CRISPLD2 augmented the expression of proinflammatory mediators in primary adult human lung fibroblast cell lines

**Figure 4:**
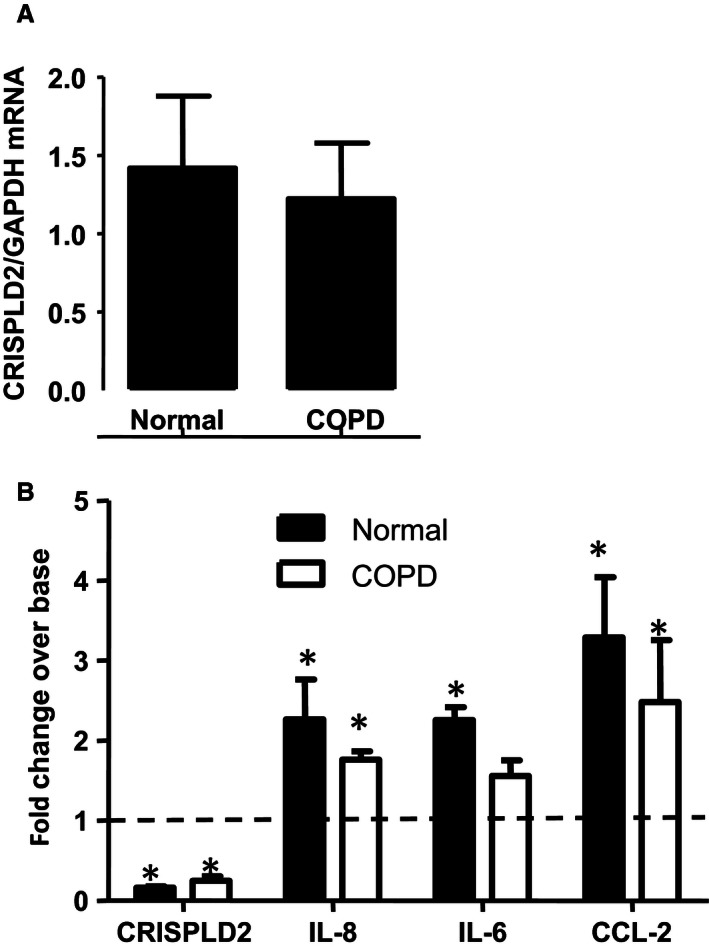
Knockdown of CRISPLD2 augmented inflammation in primary adult human lung fibroblasts. (A) endogenous expression of CRISPLD2 mRNA in primary normal and chronic obstructive pulmonary disease HLFs (*N* = 5, *P *>* *0.05). (B) CRISPLD2 was knocked down with siCRISPLD2 in HLFs (HLF^CRISPLD2siRNA^). A HLF^NCsiRNA^ control cell line was generated to be used as a control for each primary cell line. The fold change of gene expression in HLF^CRISPLD2siRNA^ over HLF^NCsiRNA^ from the same cell line are indicated in Figure 4B. CRISPLD2 expression was reduced by 80% when HLF^CRISPLD2siRNA^ cells were compared to HLF^NCsiRNA^ cells (indicated as the dot line). *IL‐8, IL6, CCL‐2* increased significantly in HLF^CRISPLD2siRNA^ (*N* = 4, *P <* *0.05, fold change of HLF^CRISPLD2siRNA^ over HLF^NCsiRNA^ from the same cells).

Initially, we characterized endogenous expression of *CRISPLD2*mRNA in five primary normal HLFs and in five primary COPD HLFs. CRISPLD2 is expressed in both normal and COPD HLF cells. There was no significant difference between the two groups in expression of CRISPLD2 (Fig. 4A), or in the expression of IL‐8 and IL‐6 (data not shown). This may have resulted from the limited number of cell lines available to us. To determine the effect of endogenous CRISPLD2 on proinflammatory mediators, we knocked down CRISPLD2 with siRNA in each primary cell line (HLF^CRISPLD2siRNA^) which was then compared to the same primary cell line treated with a nontarget control siRNA (HLF^NCsiRNA^). The fold change of gene expression in HLF ^CRISPLD2siRNA^ over HLF ^NCsiRNA^ (same cell) were measured as shown in Figure 4B. Knockdown of endogenous CRISPLD2 significantly increased mRNA expression of proinflammatory mediators IL‐8, CCL‐2, and IL‐6, in both normal and COPD adult lung fibroblast (4 normal and 4 COPD), revealing that CRISPLD2 has intrinsic antiinflammatory properties, reducing production of proinflammatory cytokines.

### RNA‐Seq transcriptome profiling of HFL^CRISPLD2siRNA^ primary cells

To identify transcriptomic changes following CRISPLD2 suppression in primary lung fibroblasts, we performed RNA‐seq expression profiling of primary HLFs from four healthy (age: 65–77 years, male) and four COPD human patients (age: 58–68 years, male, moderate‐severe COPD, GOLD classification) transfected with siRNA negative control (HLF ^NCsiRNA^) or CRISPLD2 knockdown (HLF ^CRISPLD2siRNA^), in technical duplicates. Out of 65,972 genes, 106 were significantly differentially expressed between HLF ^CRISPLD2siRNA^ and HLF ^NCsiRNA^ (Table 2). Gene ontology analysis of sets of significantly differentially expressed genes was performed using DAVID 6.7 (da Huang et al. 2009). These 106 genes were ontologically enriched for terms related to hormone stimulation response, apoptosis, focal adhesion, chemokine signaling pathway, cancer, VEGF signaling, cell development, and differentiation (Table 1). Many of these functional pathways have been previously associated with CRISPLD2 (Kaplan et al. 1999; Oyewumi et al. 2003a, 2003b; Nadeau et al. 2006; Quinlan et al. 2007; Lan et al. 2009; Wang et al. 2009, 2013; Shen et al. 2011; Yoo et al. 2014; Zhang et al. 2015). The complete set of differentially expressed genes is described in Table 2. Transcriptomic changes in these functional groups were observed in both normal and COPD lung fibroblasts in which CRISPLD2 was suppressed. There were no significant differences in the gene profile affected in the two groups.

**Table 1:** Functional annotation clusters obtained with the NIH DAVID tool using differentially expressed genes

Category	Term	Count	*P*‐value
Annotation cluster 1	Enrichment score: 2.09		
GOTERM_BP_FAT	GO:0009725 – response to hormone stimulus	9	4.86E‐04
GOTERM_BP_FAT	GO:0009719 – response to endogenous stimulus	9	9.25E‐04
GOTERM_BP_FAT	GO:0010033 – response to organic substance	11	0.003059
GOTERM_BP_FAT	GO:0032870 – cellular response to hormone stimulus	5	0.004473
GOTERM_BP_FAT	GO:0007242 – intracellular signaling cascade	12	0.047661
GOTERM_BP_FAT	GO:0048545 – response to steroid hormone stimulus	4	0.072211
GOTERM_BP_FAT	GO:0019932 – second‐messenger‐mediated signaling	4	0.114472
Annotation cluster 2	Enrichment score: 1.78		
KEGG_PATHWAY	hsa04062: Chemokine signaling pathway	7	0.00177
GOTERM_BP_FAT	GO:0032870 – cellular response to hormone stimulus	5	0.004473
GOTERM_BP_FAT	GO:0007169 – transmembrane receptor protein tyrosine kinase signaling pathway	6	0.005324
GOTERM_BP_FAT	GO:0007167 – enzyme linked receptor protein signaling pathway	7	0.007334
GOTERM_BP_FAT	GO:0008286 – insulin receptor signaling pathway	3	0.014805
GOTERM_BP_FAT	GO:0043434 – response to peptide hormone stimulus	4	0.042374
GOTERM_BP_FAT	GO:0032869 – cellular response to insulin stimulus	3	0.045857
GOTERM_BP_FAT	GO:0032868 – response to insulin stimulus	3	0.09006
KEGG_PATHWAY	hsa04914: Progesterone‐mediated oocyte maturation	3	0.123218
Annotation cluster 3	Enrichment score: 1.35		
KEGG_PATHWAY	hsa04062: Chemokine signaling pathway	7	0.00177
KEGG_PATHWAY	hsa05222: Small cell lung cancer	5	0.002729
GOTERM_BP_FAT	GO:0007169 – transmembrane receptor protein tyrosine kinase signaling pathway	6	0.005324
GOTERM_BP_FAT	GO:0007167 – enzyme linked receptor protein signaling pathway	7	0.007334
KEGG_PATHWAY	hsa04210: Apoptosis	4	0.023003
KEGG_PATHWAY	hsa04510: Focal adhesion	5	0.052017
KEGG_PATHWAY	hsa05200: Pathways in cancer	6	0.078927
KEGG_PATHWAY	hsa04370: VEGF signaling pathway	3	0.098097
KEGG_PATHWAY	hsa05210: Colorectal cancer	3	0.118536
KEGG_PATHWAY	hsa04012: ErbB signaling pathway	3	0.125576
KEGG_PATHWAY	hsa04810: Regulation of actin cytoskeleton	4	0.193564
KEGG_PATHWAY	hsa04670: Leukocyte transendothelial migration	3	0.203014
GOTERM_BP_FAT	GO:0007166 – cell surface receptor linked signal transduction	13	0.216222
GOTERM_CC_FAT	GO:0005829 – cytosol	8	0.586609
Annotation cluster 4	Enrichment score: 1.31		
KEGG_PATHWAY	hsa05222: Small cell lung cancer	5	0.002729
KEGG_PATHWAY	hsa05223: Non‐small cell lung cancer	3	0.055422
KEGG_PATHWAY	hsa05214: Glioma	3	0.072728
KEGG_PATHWAY	hsa05200: Pathways in cancer	6	0.078927
KEGG_PATHWAY	hsa05218: Melanoma	3	0.08938
KEGG_PATHWAY	hsa05212: Pancreatic cancer	3	0.091537
KEGG_PATHWAY	hsa05220: Chronic myeloid leukemia	3	0.098097
Annotation cluster 5	Enrichment score: 1.27		
GOTERM_BP_FAT	GO:0055002 – striated muscle cell development	3	0.028091
GOTERM_BP_FAT	GO:0055001 – muscle cell development	3	0.03221
GOTERM_BP_FAT	GO:0051146 – striated muscle cell differentiation	3	0.072328
GOTERM_BP_FAT	GO:0042692 – muscle cell differentiation	3	0.123692

Clusters with enrichment scores >1.0 are shown. Individual *P*‐values listed correspond to EASE Scores, or modified Fisher exact *P*‐values computed by DAVID.

**Table 2:** List of differentially expressed genes

Gene_short_name	Sig_Norm_ *P* < 0.05_2fold (strict)	RankSum_Pval_Norm	Log2Fold_Norm	Sig_COPD_*P* < 0.05_2fold	RankSum_Pval_COPD	Log2Fold_COPD
CRISPLD2	1*	0.000155	−2.21	1	0.000622	−1.77
SAMD15	1	0.000155	−1.18	1	0.000155	−1.38
RASSF8	1	0.000155	−1.36	1	0.000155	−1.15
HMGB2	0*	0.028127	−0.95	1	0.000155	−1.07
SNX4	0	0.000155	−0.97	1	0.000155	−1.12
ULBP2	1	0.020668	−1.17	1	0.020668	−1.00
ISY1	0	0.000155	−0.93	1	0.000155	−1.09
AP000275.65	0	0.046309	−0.30	1	0.001088	−1.04
GALNT4	1	0.000155	−1.26	1	0.000155	−1.07
LEPROTL1	0	0.000155	−0.91	1	0.000155	−1.03
ACTG2	0	0.000622	−0.96	1	0.001865	−1.03
LRRIQ1	0	0.000622	−0.67	1	0.000155	−1.04
TMEM67	1	0.000155	−1.09	0	0.000155	−0.99
DCP1A	1	0.000155	−1.15	1	0.000155	−1.04
POC1B‐GALNT4	0	0.38228	−0.45	1	0.028127	−1.07
KCTD9	1	0.000155	−1.06	0	0.000155	−0.98
KCTD9P4	1	0.000155	−1.16	0	0.000155	−0.82
TSC22D2	1	0.000155	−1.05	0	0.000155	−0.99
KCTD9P2	1	0.000155	−1.06	0	0.000155	−0.83
SERTAD2	1	0.004662	−1.00	0	0.000155	−0.64
RP11‐512M8.5	2*	0.004196	1.19	0	1	0.04
ZCCHC10	2	0.000155	1.01	0	0.000155	0.74
PSMB9	2	0.000311	1.04	0	0.000311	0.94
FAM26E	2	0.000155	1.02	0	0.001865	0.94
APOL3	2	0.000155	1.07	0	0.000155	0.95
SCO1	2	0.000155	1.00	0	0.000155	0.89
IL15RA	2	0.001088	1.13	0	0.000155	0.98
B3GNT5	2	0.000155	1.01	0	0.000155	0.92
SLC41A2	0	0.000155	0.71	2	0.000155	1.00
RP2	2	0.000155	1.04	0	0.000155	0.99
C3	2	0.010412	1.30	2	0.001865	1.08
MPP5	2	0.000155	1.01	0	0.000155	0.95
CXCL8	0	0.57374	0.47	2	0.010412	1.01
NBN	2	0.000155	1.12	0	0.000155	0.98
STK17B	2	0.000155	1.09	0	0.000155	0.99
SLC9A6	2	0.000155	1.05	2	0.000155	1.03
LRRC58	2	0.000155	1.22	0	0.000155	0.97
CPOX	0	0.000155	0.91	2	0.000155	1.02
CYCS	2	0.000155	1.14	2	0.000155	1.04
PCGF5	2	0.000155	1.06	0	0.000155	0.99
C12orf49	0	0.000155	1.00	2	0.000155	1.01
GPR180	2	0.000155	1.02	2	0.000155	1.04
ENPP5	0	0.049883	0.95	2	0.010412	1.11
IRAK2	0	0.001865	0.90	2	0.002953	1.01
CCL2	0	0.38228	0.87	2	0.010412	1.16
MEST	2	0.006993	1.10	2	0.000155	1.06
RP11‐554I8.2	0	0.000311	0.95	2	0.014763	1.12
ARSI	0	0.002953	0.81	2	0.004662	1.10
PIK3R3	0	0.020668	0.94	2	0.004662	1.08
SPIN4	2	0.001865	1.06	0	0.000155	0.94
CDK6	0	0.000155	0.84	2	0.000155	1.01
GNB4	2	0.000155	1.35	2	0.000155	1.11
SDPR	2	0.037918	1.22	2	0.000155	1.09
POMK	0	0.000155	0.90	2	0.000155	1.02
INIP	2	0.000155	1.13	2	0.000155	1.12
ADCY7	0	0.001865	0.94	2	0.000155	1.04
PSD3	2	0.000155	1.07	2	0.000155	1.09
AC007620.3	2	0.000155	1.49	2	0.000155	1.24
PLAG1	0	0.000155	0.91	2	0.000311	1.12
CTC‐444N24.7	0	0.000155	0.85	2	0.000155	1.01
PDCD4	2	0.000155	1.19	2	0.000155	1.19
BLOC1S6	2	0.000155	1.22	2	0.000155	1.09
PLAU	0	0.037918	0.82	2	0.000155	1.18
ENO1P4	0	0.1049	0.54	2	0.000155	1.36
KRT19	0	0.1049	0.69	2	0.049883	1.20
CTC‐444N24.11	0	0.000155	0.92	2	0.000155	1.06
RASA2	2	0.000155	1.25	2	0.000155	1.20
ZNF460	0	0.000155	0.91	2	0.000155	1.05
PMAIP1	2	0.000622	1.35	2	0.001865	1.28
SLC4A4	0	0.082984	1.11	2	0.001865	1.17
HINT3	2	0.000155	1.28	2	0.000155	1.27
ARSJ	2	0.000155	1.42	2	0.000155	1.27
EFHD2	2	0.000155	1.31	2	0.000155	1.22
ELMOD2	2	0.000155	1.50	2	0.000155	1.35
PAWR	2	0.000155	1.44	2	0.000155	1.29
PIK3R1	2	0.000155	1.23	2	0.001865	1.24
FAM46A	2	0.001865	1.29	2	0.000155	1.31
NAP1L5	2	0.000155	1.34	2	0.000155	1.39
ARL5B	2	0.000155	1.49	2	0.000155	1.32
LINC00707	0	0.037918	0.81	2	0.000155	1.21
ABCB10	2	0.000155	1.31	2	0.000155	1.34
CNOT6	2	0.000155	1.14	2	0.000155	1.25
LCLAT1	2	0.000155	1.60	2	0.000155	1.40
TBC1D5	2	0.000155	1.26	2	0.000155	1.34
HIPK2	0	0.000155	0.80	2	0.000155	1.15
C4orf32	2	0.000155	1.41	2	0.000155	1.42
RP11‐101E13.5	2	0.000155	1.46	2	0.000155	1.43
AKIRIN1	2	0.000155	1.47	2	0.000155	1.46
CPA4	2	0.000155	1.44	2	0.000155	1.50
PDHX	2	0.000155	1.67	2	0.000155	1.52
ITGB8	2	0.000155	1.34	2	0.037918	1.51
PTK2	2	0.000155	1.41	2	0.000155	1.48
SYNPO	2	0.014763	1.28	2	0.000155	1.33
SETD7	2	0.000155	1.43	2	0.000155	1.51
CAV2	2	0.000155	1.75	2	0.000155	1.59
CLDN12	2	0.000155	1.45	2	0.000155	1.56
TROVE2	2	0.000155	1.70	2	0.000155	1.57
GNPNAT1	2	0.000155	1.77	2	0.000155	1.58
TULP3	2	0.000155	1.69	2	0.000155	1.66
UBE2V2	2	0.000155	1.72	2	0.000155	1.63
AGPAT9	2	0.001865	1.53	2	0.000155	1.65
EIF4EBP2	2	0.000155	1.28	2	0.000155	1.49
LPP	2	0.000155	1.44	2	0.000155	1.51
UHMK1	2	0.000155	1.81	2	0.000155	1.76
MMP1	2	0.037918	2.11	2	0.001865	2.26
CXCL5	2	0.010256	1.89	2	0.020668	1.89
C9orf41	2	0.000155	1.90	2	0.000155	1.85

A total of 106 genes significantly differentially expressed between HLF CRISPLD2siRNA and HLF NCsiRNA (in Norm or COPD, *N* = 8, four Norm and four COPD, *P *<* *0.05 and log2 fold magnitude >1 that is, twofold). COPD, chronic obstructive pulmonary disease. *2 =  sig up in silenced / nonsilenced; *1 =  sig down in silenced/nonsilenced; *0 =  not sig

## Discussion

Fibroblasts have been recognized as emerging effector cells in chronic inflammation. They arise from monocyte precursors and preserve both the inflammatory features of macrophages and the tissue remodeling properties of fibroblasts. Fibroblasts have been implicated in the pathogenesis of chronic inflammatory states involving the lung, autoimmunity, liver, skin, and normal aging (Reilkoff et al. 2011).

In this study, we demonstrated that CRISPLD2, an antiinflammatory mediator released from pulmonary fibroblasts, directly inhibited inflammation in lung fibroblasts and epithelial cells through mesenchymal–epithelial interactions. Knockdown of CRISPLD2 in fetal lung MRC5 fibroblasts augmented levels of the proinflammatory mediator IL‐8 in LPS‐induced inflammation (Fig. 2). The protective effect of CRISPLD2 against proinflammatory mediators was also demonstrated in adult primary lung HLF cells (Fig. 4).

We previously reported that in rat fetal lung, CRISPLD2 is secreted by the fibroblasts located adjacent to the airway epithelium, and is concentrated at the tips of budding alveoli (Nadeau et al. 2006). In this study, we show that rCRISPLD2 protein has a direct effect on lung epithelial cell migration (Fig. 3A) and also inhibits LPS‐induced lung epithelial cell inflammation (Fig. 3B). Thus, the pulmonary fibroblast can contribute directly to pulmonary inflammation and ultimately to airway wall remodeling by releasing mediators that have profound effects on the surrounding environment. These mediators are interrelated and balanced to play an important role in regulating cell functions such as proliferation, migration and ECM deposition and remodeling within the lung (Chapman 2011). The IL‐6 family may promote ECM remodeling via activation of matrix metalloproteinases (MMPs) (Chapman 2011).

Our data are in accord with other findings demonstrating antiinflammatory properties of CRISPLD2. Recent studies in humans and rodents attribute antiinflammatory properties to CRISPLD2 /Crispld2. For example, Crispld2 directly binds to LPS and inhibits LPS binding to TLR4 and consequently reduces LPS‐derived inflammation. Upregulation of the serum soluble form of Crispld2 protected mice against LPS‐induced lethality (Wang et al. 2009). Low‐serum CRISPLD2 level may predict a poor outcome in patients with sepsis or septic shock (Wang et al. 2013). *CRISPLD2* knockdown increased IL‐6 and IL‐8 levels in airway smooth muscle cells induced by IL‐1*β* (Himes et al. 2014).

Cytokines can regulate cellular responses to inflammation both reversibly (e.g., synthesis/secretion of specific proteins) and irreversibly (e.g., cell division or apoptosis), through the coordinated effects of positive and negative signals. Cytokines modulate airway remodeling and fibrosis in respiratory diseases such as BPD, COPD, asthma, and interstitial lung diseases (Chapman 2011). Our present findings suggest that CRISPLD2 is involved in the regulation of endotoxin‐induced inflammatory responses of lung fibroblasts and epithelial cells, and support the attribution to CRISPLD2 of a role in airway remodeling through mesenchymal–epithelial interactions.

Our findings are consistent with earlier work demonstrating a role for CRISPLD2 in antiinflammatory signaling in the lung. We showed previously that *Crispld2* mRNA expression is upregulated in rat fetal lung fibroblasts in response to GCs (Kaplan et al. 1999; Himes et al. 2014). More recently, using RNA‐seq profiling, we (STW) identified *CRISPLD2* as a GC‐responsive gene that modulates cytokine function in airway smooth muscle cells (ASM) (Himes et al. 2014). In a search for the mechanism of action of GC‐responsive genes that suppress inflammation in asthma, Himes et al. identified CRISPLD2 as a GC target. CRISPLD2 expression was also induced by IL‐1*β* and siRNA‐mediated knockdown of CRISPLD2 increased IL‐1*β* induced expression of IL‐6 and IL‐8 in ASM cells (Himes et al. 2014). These findings suggested that CRISPLD2 may have therapeutic potential in asthma.

We found that silencing of CRISPLD2 impacted transcriptomic changes related to multiple cell functions including hormone stimulation response, apoptosis, focal adhesion, chemokine signaling pathway, cancer, VEGF signaling, cell development, and differentiation in human lung fibroblasts (Table 1). Of interest, these effects were found in normal cells as well as in cells isolated from lungs of patients with COPD. In recent work directed at identifying potential therapeutic strategies for treatment of COPD, Greer et al. investigated the potential cooperative effects of adenosine A_2B_ receptor agonists and GCs as antiinflammatory agents in BEAS 2B cells (Greer et al. 2013). Among the antiinflammatory genes induced in these cells was CRISPLD2. Our RNA‐seq results confirm the efficiency of CRISPLD2 knockdown and support the functional roles of CRISPLD2 in extracellular matrix development and function, and in inflammation. The extent of its role as an antiinflammatory mediator in COPD, however, remains unclear. Clearly, the effects of CRISPLD2 suppression may induce similar transcriptomic changes in lung fibroblasts and yet contribute to even more profound effects on the phenotype of COPD patients. It is also important to note that if CRISPLD2 has antiinflammatory effects – even if it is not a primary inducer of inflammation in COPD – it may still have potential as a broad‐spectrum therapeutic agent in the treatment of lung inflammation in COPD and other lung pathologies.

## Conclusion

Our studies support the hypothesis that suppression of CRISPLD2 increases the risk of lung inflammation in early life and in adulthood. CRISPLD2 serves as an endogenous antiinflammatory protein which can curtail proinflammatory signaling by lung epithelial cells through mesenchymal–epithelial interactions. Further investigation of the potential of CRISPLD2 to serve as a therapeutic candidate is imperative.

## Conflict of Interest

None declared.
